# Art and Disease

**Published:** 1894-02-10

**Authors:** 


### ART AND DISEASE.

### V.—THE LAME AND THE BLIND.

Early art has preserved two specimens at least of artificial supports applied to injured or amputated limbs. The first is a mosaic from the cathedral of Lescar, of uncertain but probably very early date. The mosaic is much defaced, but it reveals a negro in the act of hunting, with his bow stretched to shoot, and a gigantic horn slung at his back. His right leg has lost its foot, and is bent back so that it rests on the fork of a wooden stump attached.* The stump is said to be precisely similar to those used in the present day; the knee is supported by it, and the leg, bent at a right angle, projects behind. An ancient fragment of pottery discovered some years ago in Paris portrays a somewhat similar incident of the chase. The hunter is a very remarkable figure, entirely nude, of immense muscular force. His left arm is outstretched and the hand holds a lyre, while the right hand is raised with a wild gesture over his head. He is half resting on a kind of antique bench; the right leg has evidently been mutilated by an amputation, either natural and resulting from some kind of gangrene, or surgical, and practised below the knee. Thus the stump, which terminates the leg, slightly swollen at the level of the muscles which commence the calf, rests on a veritable drumstick of a special pattern, perfectly different to the wooden leg in the mosaic of Lescar. It is straight and cylindrical in the middle, but is enlarged at the extremities, making a sort of concave support for the injured limb.

In Christian art the introduction of the lame is frequent in pictures of miracles. It is very strange with what interest artists have dwelt on the physical peculiarities of their models, and with what care they have reproduced symptoms which might have escaped the eye of all but a practised physician. M. Charcot observes that Raphael in particular has been so much struck with the common features betraying a constitution given over to rickets, that he has almost exclusively appropriated this type in painting the lame and infirm. And it is curious that he should have taken heed that a malformation of the limbs, which seems at first sight purely a local malady, should be a symptom of an ill exhibited in other parts of the body, and clearly marked on the face. The peculiar physiognomy of rickets, "which in small children gives so curious an air of sadness and precocious old age, retains always in later life something characteristic which may be thus defined. Skull more or less deformed, forehead sometimes over prominent, sometimes low and retreating; ears large and projecting; thin, furrowed face with prominent cheek bones; hollow sockets, from the depth of which the eyes appear more brilliant; prominent jaw, and widely-stretched mouth with projecting teeth; rarely any beard."† Any-one may satisfy himself by an examination of engravings from the Cartoons, of the fidelity with which Raphael has adhered to this type. In "The Sacrifice at Lystra," although the miracle is performed and the cripple is healed, he retains in his face the marks of the malady which had made him lame. In "The Healing of the Lame," M. Charcot detects an incongruity in the limb of the principal suppliant, which suggests that Raphael, as in the case of the demoniac boy, dispensed for the occasion with a real crippled model. He has not, however, fallen into the error attributed to one of his pupils, who, in a fresco at the Vatican, has depicted a helpless invalid "with the muscles of a Hercules," and with limbs as stout as his body, and who, perfectly capable of supporting it, is dragging himself painfully along on his knees.

In studying the representations of the blind two distinct types become apparent, but the difference is marked rather by attitude than physical conformation. In the first type, in whom blindness is total and there is no perception of light, the head is represented as thrown back, the body erect, and the eye directed above as though in search of light. The well-known bust of Homer, in the Museum at Naples, conveys this sense of searching for light by the unequally opened eyes, with eye-brows raised above, and is one of the oldest specimens of an attempt to render blindness in art. But there are many affections of the eye in which the presence of light is intensely painful, and the expression is then completely changed. The eyebrows are contracted in a frown, or the lids are altogether closed, while the head is bent forward towards the ground. It is the former type which has been most readily seized by artists, and the early Italian masters show many examples where the rigid, erect figure, the impassible face and up-turned head appear to have been copied from life. In the Cartoon of Elymas, Raphael has finely rendered the attitude of one suddenly deprived of sight, turning his face instinctively towards the light, and stooping forward with outstretched heads in dread and uncertainty for some guide.

Strangely enough M. Charcot has discovered some of the most faithful studies of the blind in Japanese art. It appears that affections of the eyeball are particularly frequent and severe in Japan, and the indigent blind form a class apart, and may be seen in the evening passing from one street to another, armed with a long stick to serve as guide, and with a kind of flute on which they sound a prolonged note to give warning of their approach. The artist who has painted them most successfully is Hokosaï. In one of his pictures they are shown crossing a ford with great precaution; some are sounding the depth of the river with their feet and sticks; others are following cautiously behind holding one on to another; a young man is attending to one of the aged who is afraid to advance. Every face has its characteristic expression, and some show a considerable amount of humour, but, though some of the eyes are open, they are all unmistakably blind.

* Prothèse Chirurgicale chez les Anciens. Par E. Rivière.

† Les Malades et les Difformes dans l'Art. J. M. Charcot et P. Richer.

